# Informal care: the indispensable pillar of care for complex patients

**DOI:** 10.3325/cmj.2023.64.448

**Published:** 2023-12

**Authors:** Aleksandar Džakula, Maja Banadinović, Karmen Lončarek, Dorja Vočanec

**Affiliations:** 1 Department of Social Medicine and Organization of Health Care, Andrija Štampar School of Public Health, University of Zagreb School of Medicine, Zagreb, Croatia *dvocanec@snz.hr*; 2 PIN for Health & University of Zagreb, School of Medicine, Zagreb, Croatia (PhD student); 3 Center for Integrated and Palliative Care, University of Rijeka, Faculty of Medicine, Rijeka, Croatia

## Irreplaceability of informal care

If our civilization were to showcase its strength and prowess, few achievements would rival the impact of medical technologies. These advancements seamlessly integrate countless innovations, spanning disciplines from biotechnology to psychology, and from ethical reflection to artificial intelligence. Despite the remarkable strides in medical technologies, the essence of caring for human health remains irreplaceable without the personal touch and concern of individuals (1,2). This individual may be a seasoned professional with whom we have cultivated a close, professional partnership. However, patients desire the presence of another living person by their side, someone who tends to them not just as a professional but as a fellow human being. The concept of comprehensive patient care is challenging to envision without the essential element of human care – a seemingly simple, yet technologically irreplaceable, form of support (3).

Today, a rising challenge lies in informal care. Genuine goodwill, dedicated time, and a sincere desire to assist often fall short of providing sustainable care. To achieve lasting impact and integration with other forms of care, it is crucial that those involved in formal care comprehend the nature of informal care – its providers, means, and methods, and the need for continuous enhancement and expansion of resources and opportunities for informal care provision (4). The diversity within modern medical technology, coupled with the potential for rapid networking, has created significant opportunities for quick and comprehensive development of informal care. This adaptability allows informal care to be tailored to the specific needs and situations of individual patients or their caregivers. Moreover, contemporary technologies facilitate long-distance communication between informal caregivers and professionals, serving as an extended support system for health care professionals precisely when and where it is most beneficial for the patient (5,6). Numerous positive examples demonstrate a successful integration of informal and formal care. In practical terms, this signifies the feasibility of involving and training informal caregivers in a spectrum of activities crucial for the patient's daily life, and in the processes of treatment and rehabilitation (7).

Conversely, shifts in family size and structure, the evolving dynamics of local communities, and contemporary lifestyles pose challenges to the provision of informal care. Many patients now reside alone or in two-member families. Extended families lack consistent contact and the ability for members to provide ongoing care. Communities have become more diverse and less equipped to offer comprehensive and continuous support. This underscores the importance of establishing support and care networks from the outset, especially for patients with complex needs (8).

Current technological and social trends present a challenge in harmonizing high-tech and tightly regulated medicine with lay care, which is inherently personalized and often centered around individual patients or specific treatment episodes. Balancing these contrasting approaches becomes a complex task in the evolving health care systems. The transition from informal care toward a more formalized structure encounters various limitations. Primarily, individuals typically assume informal caregiving roles unintentionally or sporadically, which exposes them to heightened demands and stress. Professionals need to anticipate and facilitate the swift involvement of these “opportunistic” caregivers in the care process. To achieve this, mechanisms should be in place for rapid and effective training and support of informal caregivers. Importantly, they should not solely be positioned as assistants to formal caregivers but be empowered to act independently within a comprehensive and coordinated care network for the patient. By systematically connecting formal and informal care, the institutionalization of patients is minimized (3). A fundamental prerequisite for the effective integration of informal care is a professional attitude that recognizes, understands, and respects the value of informal caregiving.

## Informal care characteristics

Formal and informal care often pose challenges to integration due to their starting differences (Table 1). The key distinctions lie in the fact that formal care is paid and operates independently of social relations, whereas informal care is unpaid, and informal caregivers are related to care recipients (or dependent persons) through familial or non-familial relations. Informal care further encompasses two types: professional care provided by unregulated professionals, and lay care, which is performed by family members, friends, relatives, neighbors, or volunteers. Recognizing these differences is essential for fostering effective collaboration between formal and informal care networks (9).

**Table 1:** Basic characteristics of formal and informal care

Feature	Formal care	Informal care
Provider	Trained professionals	Family members, friends, or neighbors
Payment	Paid services	Unpaid services
Setting	Structured setting (nursing home, assisted living facility, home health agency)	Home-based or community-based
Regulation	Regulated by government agencies or professional organizations	Not regulated
Focus	Specific tasks and services	Personalized care and support
Flexibility	Schedules and services may be more rigid	Schedules and services may be more flexible
Emotional connection	May have less emotional connection with the recipient	May have a strong emotional connection with the recipient

In certain life situations, professionals may take on caregiving roles or provide care in ways that are not fully regulated or officially part of the health system. An example of this is volunteering undertaken by health care workers, labeled as “professional informal care.” In these instances, health care professionals extend their expertise beyond the formal health care framework, contributing valuable support in informal caregiving capacities.

Conversely, family members can assume formal roles in the patient's long-term care. In these cases, they receive compensation for their dedicated work in providing continuous care, and ideally undergo active education and organization. An example of formal lay care is a parent caregiver offering long-term home care for a chronically ill child, or a family caregiver providing extended care for a chronically ill adult.

## Multifaceted challenges of informal care

While the complementary nature of formal and informal care forms the foundation for comprehensive patient care, there are challenges in implementing such a care model. After all, the distinction between formal and informal care, paid and unpaid care is becoming increasingly blurred, which carries important implications for the role of informal carers and the quality of the provided care (10). The effective integration and coordination of these diverse caregiving approaches pose significant challenges for successful implementation. The complexity of relationships, coupled with the often extensive scale and duration of the care required, presents a highly demanding situation for this caregiving ecosystem. The challenges arise from the multifaceted dynamics involved in providing sustained care over a prolonged period, emphasizing the need for thoughtful strategies and comprehensive support systems within this caregiving framework.

Initially, there is the question of which and how much information the informal caregiver should know regarding the patient's condition and to what extent they are permitted to participate in the patient's decision-making process. Even in close relationships, patients may withhold certain information or keep it confidential. Additionally, considerations arise regarding the necessity of a parallel communication channel between the health care worker and the informal caregiver for confidential discussions without the patient's knowledge. Striking a balance between transparency, patient autonomy, and effective collaboration is crucial in navigating these challenges.

The frequent question arises about whether informal caregivers can, and are permitted to, operate within the premises of health institutions. Can we extend permission for informal caregivers to work in areas demanding specific hygiene standards or special safety requirements typical of modern hospitals? In essence, should there be a minimum qualification framework enabling informal caregivers to provide extended care within health institutions?

Instances where patients or their family members enlist a third person as a caregiver for a sick or elderly individual present unique challenges. Often, this arrangement involves informal employment, with the caregiver residing in the same household as the person under their care. In many developed countries, these caregivers are frequently immigrants, facing language barriers and differences in social and cultural backgrounds (11).

In less developed countries, the scenario is markedly different. Informal caregiving is frequently taken over by family members, predominantly women. In these situations, female caregivers often find themselves overwhelmed by their roles. Whether caring for their child, spouse, or a member of their own or their husband's family, this noble and crucial responsibility can lead to social deprivation, a decline in their own health, or financial dependence on the individual they are caring for (12). Furthermore, caregiver burnout manifests itself in a vicious circle - declining motivation to work results in an inability to sustain the necessary level of care, further diminishing motivation and perpetuating the cycle. Communication issues and various conflicts involving both caregivers and patients may arise.

## In conclusion…

Considering all the factors mentioned, informal care encounters challenges akin to those faced by modern medicine. These challenges encompass the use of modern communication technologies, advanced aids, and artificial intelligence, as well as bioethical dilemmas, and concerns related to social justice. It is essential to thoroughly study both the positive and negative aspects of such experiences before conducting a needs analysis in any given environment. Tailoring support measures to the unique circumstances and challenges faced by informal caregivers is crucial for developing effective and targeted assistance programs.

Regardless of the magnitude of these challenges and the complexity involved in resolving them, informal care remains irreplaceable. A scenario in which a person is denied informal care is a civilizational reach, but unfortunately a negative one. In such a scenario, the complex patient becomes notably vulnerable. Informal caregivers, in this context, play a crucial role by providing flexible and comprehensive care for complex patients in at least two ways. First, they step in during resource shortages, filling critical gaps. Second, they contribute to the essential adaptation to the specific needs of a complex patient through their active involvement in care.

While the absence of care from another human being not exclusively bound by a professional relationship may not signify the end of the world for a sick person, it certainly signifies the end of the world we aspire to create – a world where supported informal care collectively contributes to a holistic and supportive environment for those facing health challenges.
